# Characterization and Antioxidant Activity of Released Exopolysaccharide from Potential Probiotic *Leuconostoc mesenteroides* LM187

**DOI:** 10.4014/jmb.2103.03055

**Published:** 2021-06-10

**Authors:** Qing Zhang, Jie Wang, Qing Sun, Shu-Ming Zhang, Xiang-Yang Sun, Chan-Yuan Li, Miao-Xin Zheng, Wen-Liang Xiang, Jie Tang

**Affiliations:** Key Laboratory of Food Biotechnology, College of Food and Bioengineering, Xihua University, Chengdu 610039, Sichuan, P.R. China

**Keywords:** Characterization, released exopolysaccharide, *Leuconostoc mesenteroides*, antioxidant

## Abstract

A released exopolysaccharide (rEPS)-producing strain (LM187) with good acid resistance, bile salt resistance, and cholesterol-lowering properties was isolated from Sichuan paocai and identified as *Leuconostoc mesenteroides* subsp. mesenteroides. The purified rEPS, designated as rEPS414, had a uniform molecular weight of 7.757 × 10^5^ Da. Analysis of the monosaccharide composition revealed that the molecule was mainly composed of glucose. The Fourier transform-infrared spectrum showed that rEPS414 contained both α-type and β-type glycosidic bonds. ^1^H and ^13^C nuclear magnetic resonance spectra analysis showed that the purified rEPS contained arabinose, galactose, and rhamnose, but less uronic acid. Scanning electron microscopy demonstrated that the exopolysaccharide displayed a large number of scattered, fluffy, porous cellular network flake structures. In addition, rEPS414 exhibited strong in vitro antioxidant activity. These results showed that strain LM187 and its rEPS are promising probiotics with broad prospects in industry.

## Introduction

Lactic acid bacteria (LAB) are the principal source of probiotic strains and are part of the natural flora in the human gastrointestinal environment [1]. Based on their long history of use in a wide array of food products, they are regarded as safe according to the Generally Recognized As Safe list from the U.S. Food and Drug Administration [2]. As one of the most important functional components of LAB metabolic products, exopolysaccharides (EPS) are economically important because they can impart positive functional effects to food and benefit health [3, 4]. In recent years, EPS have been found to possess numerous beneficial biological effects, including anti-inflammatory [5, 6], antioxidant [7, 8], immunomodulatory [9, 10], and antitumor activities [11, 12].

EPS can be classified as homopolysaccharides or heteropolysaccharides according to whether they are composed of one or more types of sugars [13]. Structural factors such as molecular weight, monosaccharide composition, and chemical groups are widely accepted determinants of EPS physiological activities [14]. Therefore, the search for novel EPS-producing strains with ideal functional characteristics has become a focus of current research [15].

Sichuan paocai, or Chinese pickle, is a widely used traditional Chinese food that is rich in LAB, which are the dominant microorganisms under fermentation conditions [16]. Liu *et al*. [17] screened an EPS-producing strain of *Lactobacillus plantarum* HY isolated from Sichuan paocai, and purified HY EPS showed considerable antioxidant and alpha-amylase inhibitory activities. In addition, Hu *et al*. [18] screened a strain of *Bacillus* sp. S-1 from Sichuan paocai, and the EPS produced by this strain was found to effectively prevent oxidative damage. *Leuconostoc mesenteroides*, the most widely distributed type of LAB, has been implicated in human health [19]. However, few studies have focused on *L. mesenteroides* isolated from Sichuan paocai.

In this study, we screened 13 released EPS (rEPS)-producing strains of LAB from Sichuan paocai, determined the rEPS-producing ability of the strains by using the phenol-sulfuric acid method, and identified strain LM187 as showing the highest rEPS yield. In addition, the strain showed good acid and bile salt resistance, and may lower cholesterol. To explore the potential applications of rEPS, we isolated and identified the rEPS produced by strain LM187 and investigated their antioxidant activities in vitro.

## Materials and Methods

### Microorganism and Culture Conditions

Strain *L. mesenteroides* LM187 was isolated from a sample of traditional Sichuan paocai and showed the highest yield among 13 strains with the ability to produce rEPS. MRS liquid medium was used for the activation and cultivation of strain LM187. rEPS was produced in sugar production medium (180 g of sucrose, 10 g of peptone, 5 g of dipotassium phosphate, and 2 g of sodium chloride). The medium for selecting strain LM187 was MRS selection medium, which was prepared with the same components as MRS except that the glucose quantity was changed to 5 g and 50 g sucrose was added. MRS-Oxgall-CHOL (MOC) medium was used to measure the cholesterol-lowering ability of strain LM187 and was prepared with the same components as MRS except that 100 μg/ml water-soluble cholesterol was added, followed by sterilization at 121°C for 15 min.

### Identification of Strain LM187

Strain LM187 was successively identified by colony morphology, Gram staining, cell morphology, physiological and biochemical tests, and 16S rRNA sequence analysis. The 16S rRNA gene was amplified by PCR using primers EU27F (5′-AGAGTTTGATCCTGGCTCAG-3′) and 1492R (5′-GGTTACCTTGTTACGACTT-3′). Thermal cycles were as follows: 95°C for 5 min, 30 cycles of 94°C for 1 min, 50°C for 1 min, and 72°C for 2 min, and 72°C for 10 min. The 16S rRNA sequences were compared with those in the GenBank Nucleotide Library for sequence similarity using BLAST software (https://blast.ncbi.nlm.nih.gov/Blast.cgi). A phylogenetic tree was analyzed and constructed by the neighbor-joining method using MEGA version 5.0 software.

### Bioactivities of Strain LM187

According to a report by Vinderola *et al*. [20], the pH of the human stomach is typically maintained in the range of 3.0–5.0. Therefore, in the current study, the acid resistance capability of strain LM187 was assessed. The strain was cultivated at 30°C until the cell concentration reached 1 × 108 colony-forming units (CFU)/ml, and the prepared cultures (2.0%, v/v) were inoculated into 100 ml MRS broth at various pH (2.0, 2.5, 3.0, 3.5, 4.0, 5.0, or 6.86) and incubated at 30°C for 8 h. The viable bacterial count was determined according to GB4789.2-2016, and the survival rate was calculated as follows:

Survival rate (%) = (logN_1_/logN_0_) × 100%,

where N_1_ is the number of viable bacterial colonies after acid-resistant treatment and N_0_ is the number of viable colonies treated with MRS liquid medium at pH 6.86.

The bile salt resistance of strain LM187 was determined by inoculating the culture (2.0%, v/v) into MRS medium containing different bile salt concentrations from 0% to 0.3%, followed by incubation at 30°C for 12 h and colony counting.

To examine the cholesterol-lowering ability of LM187 [21], mixtures of 2, 5, 10, 12, 15, or 20 μg/ml cholesterol alcohol solution and MRS liquid medium were prepared. The total cholesterol concentration in the supernatant was determined using the O-phthaldehyde method [22]. Prepared cultures of the strain (2.0%, v/v) were inoculated into 10 ml MOC medium and incubated at 30°C for 12 h. The control sample contained uninoculated MOC medium, and the ability of the strain to clear cholesterol was calculated according to the standard curve, y = 0.0354x − 0.0141 (R^2^ = 0.999) as follows:

Cholesterol clearance rate (%) = [(C_0_ − C_1_)/C_0_] × 100%,

where C_0_ is the initial cholesterol content and C_1_ is the cholesterol content of the supernatant.

### Preparation and Purification of rEPS

Fresh sugar production medium was inoculated (2%, v/v) with LM187 culture suspension and incubated at 30°C for 36 h. rEPS was prepared as described by Savadogo *et al*. [23] with some modifications. After bacterial growth, the cells were pelleted by centrifugation at 10,000 ×*g* for 10 min at 4°C, and the cell-free supernatant was precipitated by mixing with a three-fold volume of absolute cold ethanol. The crude rEPS was allowed to stand overnight at 4°C, after which the sample was centrifuged at 10,000 ×*g* for 15 min. This step was repeated several times. rEPS in strain LM187 was quantified by the phenol sulfuric acid method using glucose as a standard, which was detected at 490 nm, and a standard curve was used to calculate EPS content [24]. The standard curve was y = 0.0073x + 0.0591 (R^2^ = 0.9979).

rEPS was purified using the method described by Wang *et al*. [25] with some modifications. Papain solution (0.2 ml, 10 mg/ml) was added to the crude rEPS solution, the pH was adjusted to 6.5, and the solution was placed in a 55°C water bath for 4 h. Next, 4% (w/v) Sevag solution was added to remove the proteins. The precipitated rEPS was collected by centrifugation and maintained overnight. The resulting rEPS was dried at 90°C, and the precipitate was dissolved in distilled water. Finally, the solution was dialyzed (cut-off 3500 Da) against distilled water and freeze-dried. Purified rEPS was used for subsequent analysis [26].

### Characterization of rEPS Fractions

**UV and infrared spectra of rEPS.** The rEPS (0.1 mg/ml) was dissolved in distilled water and scanned from 200 to 400 nm with a UV spectrophotometer 2400 (Shanghai Sunny Hengping Scientific Instrument Co., Ltd., China) to determine the purity of the sample.

The main functional groups of the rEPS were detected by Fourier transform-infrared spectroscopy (FT-IR) [27]. Purified rEPS powder (2 mg) was mixed with dry potassium bromide for FT-IR recording in the wavelength range of 4,000–400 cm^-1^.

**Determination of molecular weight (Mw) of rEPS.** The molecular weight and molecular weight distribution of rEPS414 were measured by gel permeation chromatography multiangle laser light scattering (GPC-MALLS). The mobile phase was NaN_3_ (0.02%) at a flow rate of 1.0 ml min-1, and the column temperature was 40°C. The injection volume was 20 μl.

### Monosaccharide Components Analysis

The monosaccharide composition of the rEPS was determined by pre-column derivatization high-performance liquid chromatography (HPLC) [28]. The purified sample (5 mg) was dissolved in 0.5 ml of 4 M trifluoroacetic acid and heated at 120°C for 2 h to hydrolyze the EPS into its component monosaccharides under a nitrogen atmosphere. The hydrolyzed sample of the purified rEPS fraction was dissolved in 0.5 ml of 0.3 M aqueous NaOH and 0.5 M PMP (dissolved in methanol) and incubated for 1 h at 70°C. After cooling to 25°C, 0.5 ml of 0.3 M HCl was added to neutralize the mixture. The resulting solution was extracted with chloroform (0.5 ml), and the process was repeated three times. The aqueous layer was filtered through a 0.45-μm membrane and subjected to HPLC analysis. Standard monosaccharides were also pre-treated according to the above steps [2].

HPLC analysis of 1-phenyl-3-methyl-5-pyrazolone (PMP) monosaccharides was carried out on an HPLC system equipped with a UV-Vis detector. Mobile phase A was 0.1 M potassium dihydrogen phosphate (pH 6.9), and mobile phase B was acetonitrile. Elution was carried out with isocratic elution using 82% mobile phase B at a flow rate of 1.0 ml/min at 25°C. The injection volume was 10 μl, and the wavelength for UV detection was 245 nm. The analytical column used was a SHISEIDO C18 (4.6 × 250 mm, 5 μm; Japan).

### Nuclear Magnetic Resonance Analysis

Purified EPS (50 mg) was dissolved in 1.0 ml D_2_O and analyzed. High-resolution ^1^H and ^13^C nuclear magnetic resonance (NMR) spectra were recorded on an NMR spectrometer operating at a sample temperature of 25°C.

### Scanning Electron Microscopy Analysis

Scanning electron microscopy (SEM) images of purified rEPS samples were obtained after fixing the samples onto aluminium stubs and gold-sputtering. A Hitachi SU8010N field emission scanning electron microscope (Japan) was used to observe and record the representative visual field.

### Assay of Antioxidant Activities

**DPPH free radical scavenging assay.** DPPH free radical scavenging activity was measured as described by Rai *et al*. [29]. Purified rEPS was dissolved in water at various concentrations (0.2, 0.4, 0.6, 0.8, and 1.0 mg/ml^-1^). Next, 2.0 ml of each sample was mixed with 2.0 ml of 0.2 M DPPH-ethanol solution. The mixture was shaken well and left for 30 min at 25°C in the dark, and then measured at 517 nm in a UV spectrophotometer. The same concentration of ascorbic acid (vitamin C, Vc) was used as a control. The DPPH radical scavenging capability was calculated using the following formula:

Scavenging activity (%) = [1 − (A_1_ − A_2_)/A_0_] × 100%,

where A_0_ is the absorbance of a mixture of 2 ml DPPH and 2 ml distilled water, A_1_ is the absorbance of a mixture of 2 ml DPPH and 2 ml purified rEPS solution, and A_2_ is the absorbance of a mixture of 2 ml purified rEPS solution and 2 ml ethanol.

**Hydroxyl radical scavenging activity.** The hydroxyl radical scavenging activity was calculated as described by Xia [30] with some modifications. Purified rEPS was dissolved in water at various concentrations (0.2, 0.4, 0.6, 0.8, and 1.0 mg ml^-1^). Next, 2.0 ml of each sample solution was mixed with 2.0 ml of 6 mM FeSO_4_ and H_2_O_2_ solution. The mixture was shaken well and left for 10 min, and 2.0 ml of 6 mM salicylic acid was added. The mixture was left for 40 min, and the absorbance was measured at 510 nm. The same concentration of ascorbic acid was used as a control.

Scavenging activity (%) = [1 − (A_1_ − A_2_)/A_0_] × 100%,

where A_0_ is the absorbance of distilled water, A_1_ is the absorbance of the sample, and A_2_ is the absorbance of the sample without H_2_O_2_ solution.

**Superoxide radical scavenging activity.** The scavenging activity of superoxide anion free radicals was determined by the pyrogallol auto-oxidation method [31]. Each sample solution (0.1 ml) was mixed with 4.5 ml of 0.05 M Tris-HCl (pH 8.2). The mixture was shaken well and left for 10 min at 25°C. Next, 0.4 ml of 10 mM pyrogallol solution was added to the mixture and left for 25 min. The reaction was stopped by adding 300 μl of concentrated HCl, and the absorbance was measured at 320 nm. The same concentration of ascorbic acid was used as a control. Scavenging activity was determined as follows:

Scavenging activity (%) = [1 − (A_1_ − A_2_)/A_0_] × 100%,

where A_0_ is the absorbance of distilled water in the mixture rather than in the sample, A_1_ is the absorbance of the sample, and A2 is the absorbance of distilled water in the mixture rather than the pyrogallol.

### Statistical Analysis

All experiments were repeated three times and the results represented by their mean ± SD; *p* < 0.05 was considered statistically significant. Data were analyzed using SPSS 19.0 software (SPSS, Inc., USA).

## Results and Discussion

### Identification of Strain LM187

**Fig. 1:**
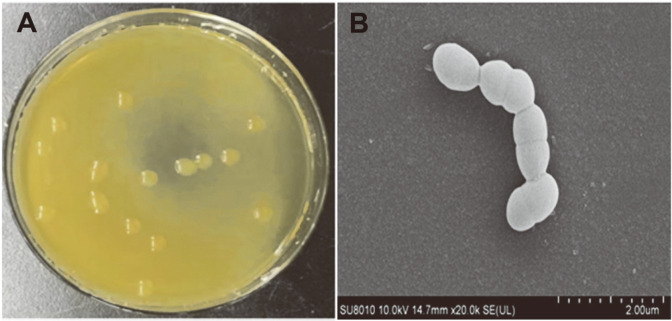
Colony and cell morphological characteristics of strain LM187.

**Fig. 2:**
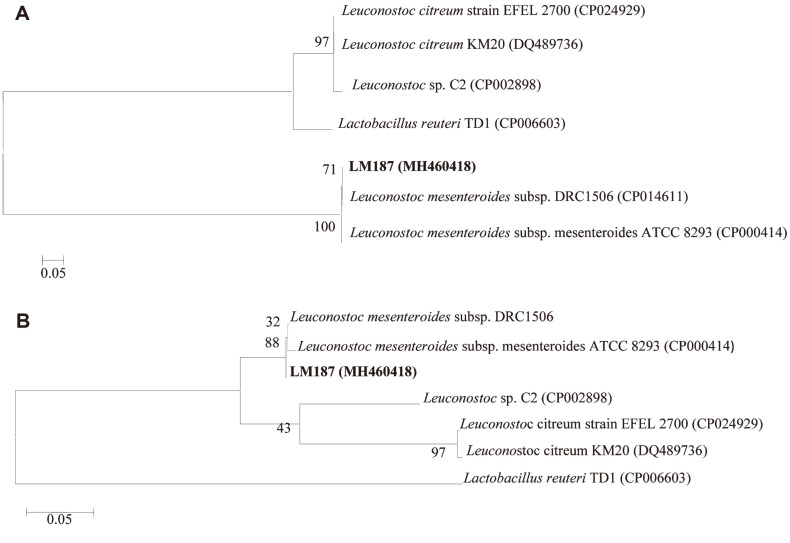
Phylogenetic tree of strain LM187 based on 16S rRNA (A) and *rpoB* (B) sequences.

Lactic acid bacteria were screened from Sichuan paocai samples, and selected strains were inoculated on sugar production medium agar and cultured at 30°C. Among 13 strains found to produce rEPS, strain LM187 showed the highest yield. The colony and morphological characteristics of LM187 are shown in Fig. 1. Analysis of the colony morphology (Fig. 1A) showed that the strain formed mucoid colonies on the surface of the medium, which were milky white, shiny, and had neat edges. The cells were observed as pairs or chains of cocci under a microscope (Fig. 1B), and the strain was gram-positive. Combined with physiological and biochemical tests and 16S rRNA gene phylogeny analysis (Fig. 2A), strain LM187 was suggested to belong to *L. mesenteroides*. However, studies have shown that 16S rRNA gene sequences often show low resolution when distinguishing related species [32]. When analyzing the genetic relationship of relatives within the genus, *rpoB* housekeeping genes give higher resolution than 16S rRNA [33]. Comparative sequencing of the 16S rRNA and *rpoB* housekeeping genes allowed for successful identification of strain LM187 as *L. mesenteroides* subsp. mesenteroides (Fig. 2B) (Collection number: CGMCC 7.363, GenBank accession number: MH460418).

### Probiotic Properties of Strain LM187

**Fig. 3:**
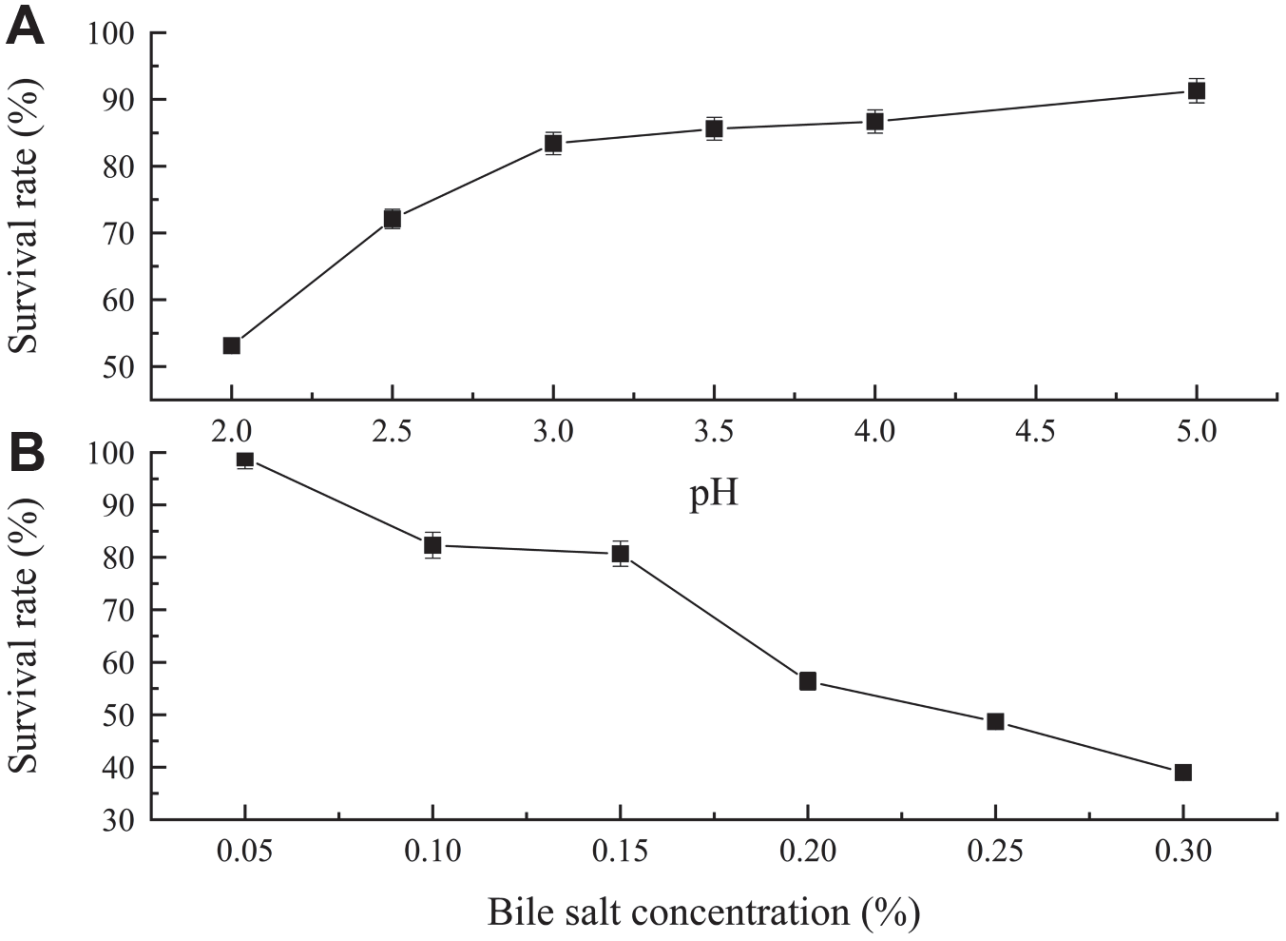
Acid and bile salt tolerance tests of strain LM187.

Microorganisms used as probiotics must overcome the inhospitable conditions of the human gastrointestinal tract [34]. To reach the gastrointestinal tract while maintaining sufficient viability, probiotic strains should be able to withstand a low pH and high bile salt concentrations [34, 35]. The results of the acid resistance test of strain LM187 are shown in Fig. 3A. After 8 h incubation, the strain survival rate was 53.1% (pH 2.0), indicating that LM187 can tolerate a low pH. These results agree with the finding that the survival rate of all LAB is significantly affected by low acidity, particularly incubation at pH 2 [34, 36]. Bile salt tolerance is considered as an essential property of LAB in the intestinal tract [37]. The bile salt resistance of this strain is shown in Fig. 3B. The strain survival rate was 39.0% when the bile salt concentration was 0.3%, indicating strain LM187 can tolerate high concentrations of bile salts. The results for bile salt tolerance observed in this study are consistent with those reported by Nami *et al*. [38], who determined the survival rate of selected LAB isolated from dairy samples.

Another important characteristic of probiotic strains is their cholesterol-lowering ability. Our findings showed that the cholesterol-lowering rate of *L. mesenteroides* subsp. mesenteroides LM187 was 53.0%. Similarly, Lee *et al*.[39] screened a strain of *L. mesenteroides* MKSR from Korean fermented vegetable kimchi, revealing a cholesterol lowering rate of 59%. Probiotic EPS has been reported to possess hypocholesterolemic bioactivities [40]. There was an earlier report that certain EPS-producing probiotics strains could bind free bile acids thus increasing their excretion after digestion and result in the synthesis of new bile acids from cholesterol by the liver and thereby decreasing cholesterol levels [21]. Hence, rEPS-producing LM187 shows potential as a probiotic based on its comparable cholesterol-lowering ability.

### UV and FT-IR Spectral Analysis of rEPS

Compared with the UV spectra of crude EPS, the UV spectra of purified rEPS showed no obvious absorption at 260 or 280 nm, clearly indicating that rEPS414 did not contain nucleic acids or proteins.

**Fig. 4:**
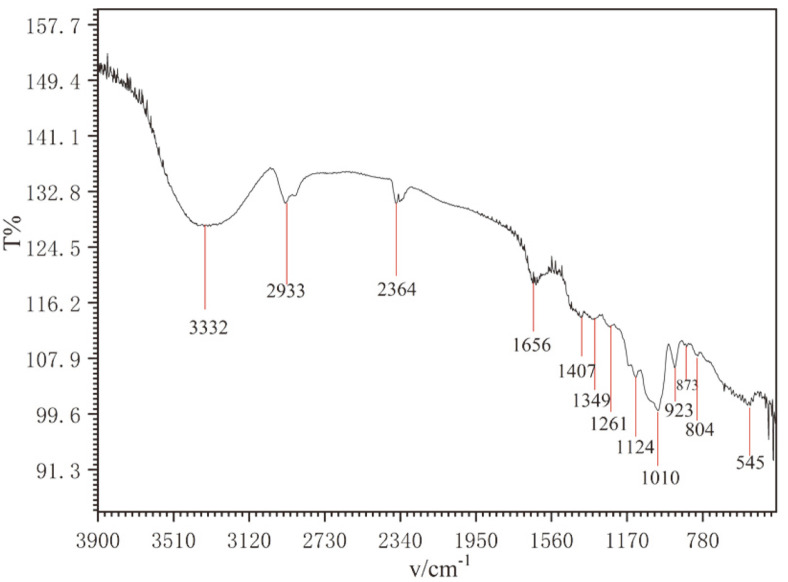
FT-IR spectrum of rEPS414.

The FT-IR spectra of purified rEPS were recorded over an absorbance range of 4,000–400 cm^-1^ and are shown in Fig. 4. Purified rEPS showed a broad absorption peak centered at around 3,332 cm^-1^, which was attributed to the intense hydroxyl group (O-H) stretching vibration [41]. The absorption peak at 2,933 cm^-1^ was assigned to the C-H bond, and weak absorption near 1,656 cm^-1^ was attributed to C=O stretching vibration [41, 42]. The strong absorption peak in the range of 1,410–1,200 cm^-1^ was dominated by the variable angle vibration of the C-H bond. Two strong absorption peaks were observed at 1,200–1,000 cm^-1^, which are characteristic absorption peaks of α-pyranosides [25]. The weak peak at around 873 cm^-1^ suggested a β-D-glucan, and a weak absorption peak at 804 cm^-1^ in the anomeric region (950–700 cm^-1^) suggested the existence of mannose [21].

### Determination of Molecular Weight

The molecular weight (Mw) and Mw distribution index were determined using a GPC-MALLS. The gel permeation chromatography result (Table 1) showed that the average Mw of rEPS414 was calculated to be 7.757 × 10^5^ Da, in agreement with the previously reported molecular weight range of LAB EPS (10^4^–10^6^ Da) [43]. The Mw distribution index was estimated to be 1.571, indicating the molar mass distribution of rEPS414 was relatively uniform. Previous studies showed that the biological activity of EPS is affected by its Mw and within a certain range; EPS of low and medium Mw appear to have stronger antioxidant and antitumor activities [11, 44].

### Monosaccharide Composition of rEPS

The monosaccharide composition of rEPS414 was analyzed by HPLC. The peaks of pre-column derivatives of ten standard monosaccharides were determined in the order of mannose (11.669 min), ribose (14.950 min), rhamnose (15.583 min), glucuronic acid (17.685 min), D-galacturonic acid (20.053 min), glucose (23.242 min), galactose (26.249 min), xylose (27.739 min), arabinose (28.768 min), and fucose (32.911 min). Compared with the monosaccharide standards, purified rEPS was mainly composed of glucose. Small amounts of galactose, rhamnose, mannose, ribose, arabinose, and galacturonic acid were found in rEPS, and no fructose was detected. Previous studies have suggested that EPS with higher glucose contents have strong antitumor activity [45].

### 1D and 2D NMR Spectroscopy

**Fig. 5:**
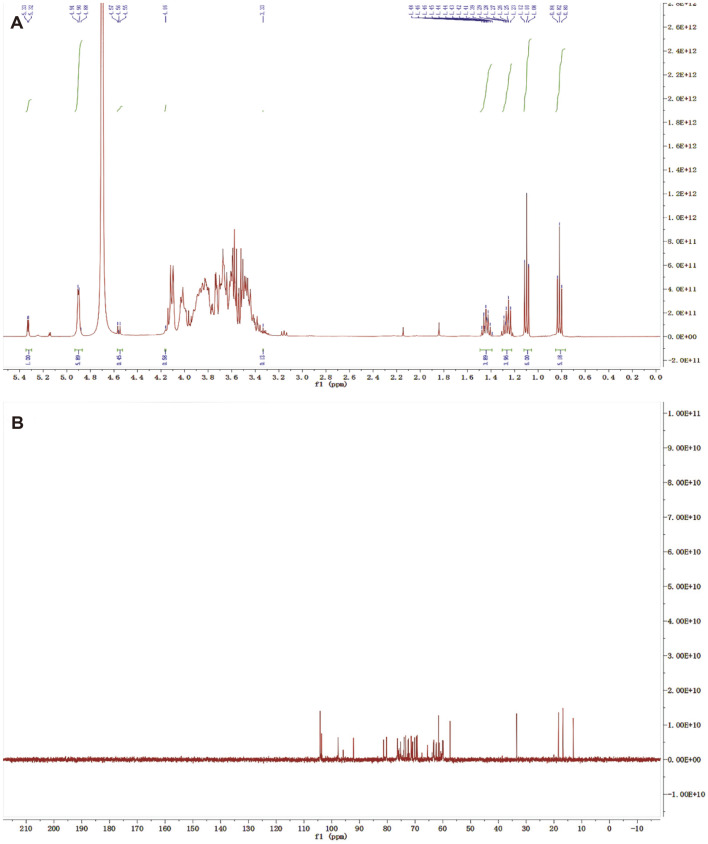
^1^H NMR (A) and ^13^C NMR (B) spectra of rEPS414.

The structural features of purified rEPS were further identified and analyzed by ^1^H NMR and ^13^C NMR. In the ^1^H NMR spectrum (Fig. 5A), anomeric proton signals were observed in downfield regions (δ 3.0–5.5 ppm), confirming the presence of polysaccharides. Fig. 5A shows that the integral ratios of the two regions of δ 4.8–5.6 and δ 3.3–4.2 ppm may contain multiple monosaccharide structures, indicating that the rEPS is a heteropolysaccharide, which is consistent with the monosaccharide composition and FT-IR analysis results. In the ^1^H-NMR spectrum, the α-type configuration generally appears at δ 4.8–5.6 ppm and β-type configuration appears at δ 4.4–4.8 ppm [46]. Hence, the signals at δ 5.33, δ 5.32, δ 4.91, δ 4.90, and δ 4.88 ppm corresponded to the α-glycosidic bond [47], whereas the signals at δ 4.57, δ 4.56, and δ 4.55 ppm corresponded to the β-glycosidic bond. The proportion of α-pyranose was much higher than that of β-pyranose, indicating that the rEPS sample contained mainly α-type glycosidic bonds. The strong peak at δ 4.70 ppm was attributed to hydrogen-deuterium oxide, and other hydrogen signals were concentrated in the range of δ 3.33–4.16 ppm and could not be resolved properly because of their overlapping chemical shifts. Furthermore, the signal at δ 1.2 ppm corresponded to the terminal methyl, but it may have been pigment interference [48].

The chemical shift of the ^13^C NMR spectrum was wider than that of the ^1^H NMR spectrum and showed less overlap, giving higher resolution. In the ^13^C NMR spectrum (Fig. 5B) of rEPS414, the region of δ 96–110 ppm relfected the sugar end group chemical shift signal region, indicating that the sample contains α-glucopyranose (δ 96–101 ppm) residues and β-glucopyranose (δ 101–110 ppm) residues. The resonance peaks of other carbon atoms were concentrated at the region of δ 60–87 ppm, and the peak at δ 82.31 ppm was due to the presence of arabinose [49]. The peaks at δ 60–80 ppm corresponded to galactose [50]. There was no obvious signal in the carboxylic signal region (δ 170–180 ppm), indicating that the sample contains less uronic acid and glycoprotein [51], which is consistent with the UV and FT-IR analysis results. The signal in the region of δ 15–17 ppm corresponds to the terminal methyl, or may be pigment interference [49]. Furthermore, a carbon signal appeared at δ 13 ppm, indicating that the rEPS sample contained rhamnose, which is consistent with the results of analyzing the monosaccharide composition.

### Microcosmic Morphology Analysis of rEPS

**Fig. 6:**
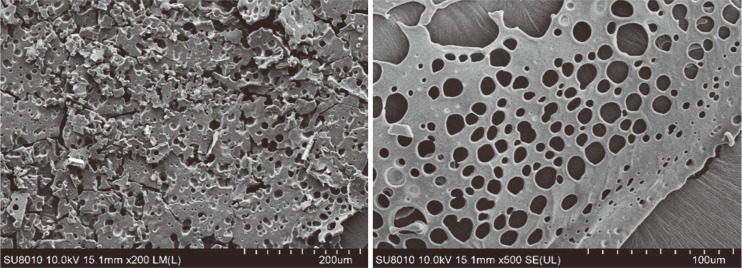
Scanning electron microscopy images of rEPS414.

As shown in Fig. 6, the SEM images of rEPS414 revealed a large number of scattered, fluffy, porous cellular network flake structures. This porous morphology indicates that the rEPS is beneficial for the diffusion of substances and metabolites required for cell growth. Its structure also showed that it has broad development and application prospects in terms of moisture retention and fragrance adsorption.

### Antioxidant Activity of rEPS

**Fig. 7:**
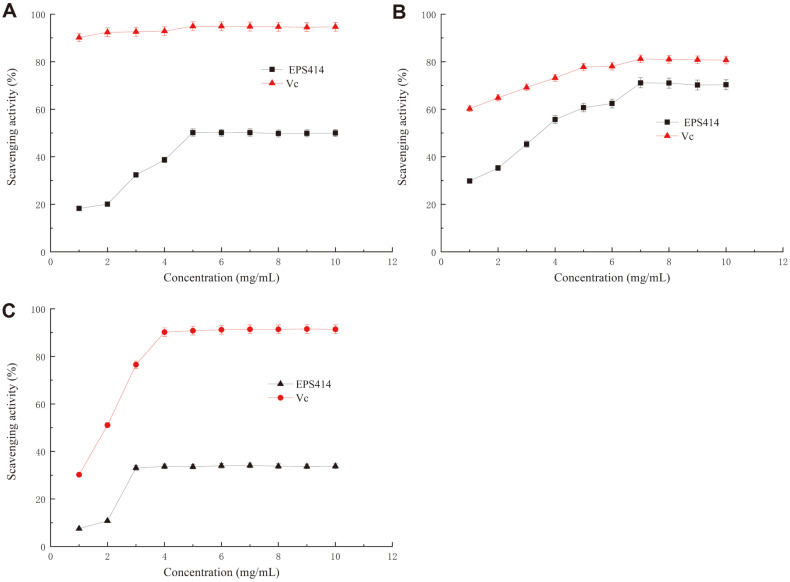
In vitro antioxidant activity of rEPS414 from *L. mesenteroides* subsp. mesenteroides LM187 using ascorbic acid (Vc) as reference. (**A**) DPPH radical scavenging activity, (**B**) Hydroxyl radical scavenging activity, (**C**) Superoxide radical scavenging activity.

DPPH radical has been widely used to determine the free radical scavenging activities of primary antioxidant activity; DPPH can accept electron or hydrogen radical to become a stable molecule [52]. The scavenging capacity of the rEPS sample for the DPPH free radical is shown in Fig. 7A; its scavenging capacity increased with increasing concentration in the range of 0–5 mg/ml. When the concentration reached 5 mg/ml, the rEPS and ascorbic acid scavenging activity reached maximum values of 50.2% and 94.9%, respectively. Bomfim *et al*. [53] evaluated the antioxidant capacity of an EPS produced by L. plantarum CNPC003, revealing a DPPH scavenging activity of 40.72% at a concentration of 4 mg/ml. Adesulu-Dahunsi *et al*. [8] also evaluated the antioxidant capacity of an EPS produced by *Weissella cibaria* GA44 and found DPPH scavenging activity of 48.9% at a concentration of 4 mg/ml. Previous studies showed that the antioxidant activity of EPS is related to its molecular weight, monosaccharide composition, and glycoside type [54]. Furthermore, impurities such as proteins and pigments in EPS can influence the antioxidant activity [55]. Thus, the specific relevance of the DPPH radical scavenging activity of the rEPS requires further analysis.

Hydroxyl free radicals are the most reactive oxygen free radicals and can react with a variety of biological macromolecules in living cells in various manners, such as by inducing lipid peroxidation and causing DNA damage. Therefore, scavenging hydroxyl free radicals is an effective approach for preventing oxidative damage to cells and tissues [56]. Some EPS isolated from LAB (such as lactobacillus) were reported to have good hydroxyl free radical scavenging activities [57, 58]. As shown in Fig. 7B, the scavenging activities of rEPS414 against hydroxyl radicals gradually increased with the concentration of polysaccharides in the range of 0–10 mg/ml, but its scavenging activities were lower than that of ascorbic acid. At a concentration of 7 mg/ml, the rEPS sample scavenging activity reached a maximum value of 71.0%, which is close to that of Vc (81.0%). Wang *et al*. [25] evaluated the antioxidant capacity of an EPS produced by *Lactobacillus fermentum* and observed a lower hydroxyl scavenging activity of 61.67% at a concentration of 4 mg/ml compared to that found in the present study. These results suggest that the rEPS has good hydroxyl radical scavenging activity. Previous studies reported that the scavenging activity of EPS for hydroxyl radicals occurred via inhibition of hydroxyl radical generation by chelating Fe^2+^ or Cu^+^, which are powerful pro-oxidants with important roles in catalytic oxidation reactions [59, 60].

Superoxide radicals can react with biological macromolecules and cause tissue damage. They can also promote the generation of other reactive oxygen species, such as hydrogen peroxide, hydroxyl radical, and singlet oxygen, which induce strong oxidative toxicity to cells [60, 61]. As shown in Fig. 7C, at different concentrations of 0–3 mg/ml, the purified rEPS displayed varying degrees of antioxidant activity in a dose-dependent manner, similar to its scavenging activity trend for DPPH free radicals. However, there was no obvious concentration dependence in the range of 3–10 mg/ml. At a concentration of 3 mg/ml, the EPS scavenging activity reached a maximum value of 33.1%, but the scavenging activities of the rEPS were lower than that of ascorbic acid. Scavenging capacity may be ascribed to the direct contact of EPS molecules with superoxide radical to form stable radicals, which may terminate the radical chain reaction [62].

The results of antioxidant activity analysis in vitro demonstrate that rEPS414 from *L. mesenteroides* subsp. mesenteroides LM187 has potential to be developed as a natural antioxidant or functional food additive.

## Conclusion

In the present study, a rEPS-producing strain (LM187) isolated from Sichuan paocai demonstrated a strong ability to survive under acid and bile salt conditions and to lower cholesterol. Purification, structural characterization, and in vitro antioxidant activities of rEPS from strain LM187 were analyzed. The results show that strain LM187 can produce rEPS and has potential as a probiotic. rEPS produced by *L. mesenteroides* subsp. mesenteroides LM187 is mainly composed of glucose with an average Mw of 7.757 × 10^5^ Da. The purified rEPS exhibited a typical polysaccharide absorption pattern and displayed a strong ability to scavenge radicals. These results indicate that strain LM187 is a promising probiotic for use in industry.

## Figures and Tables

**Table 1:** The results of rEPS414 by GPC-MALLS.

Name (g/mol)	Molecular weight (Da)	Range (g/mol)	Molecular weight distribution (%)
Molecular weight distribution index (Mw/Mn)	1.571	189000.0-3000000.0	16.2
Dispersion (Mz/Mn)	3.328	3000000.0-9500000.0	45.0
Number average molecular weight (Mn)	4.937 × 10^5^	9500000.0-15000000.0	35.4
Peak molecular weight (Mp)	3.302 × 10^5^	15000000.0-63335316.0	3.0
Weight average molecular weight (Mw)	7.757 × 10^5^		
Z average molecular weight (Mz)	1.643 × 10^6^		
